# A 25-year trend in gastrointestinal cancers in northern Iran (1991-2016)

**DOI:** 10.22088/cjim.10.4.396

**Published:** 2019

**Authors:** Hakimeh Mehdizadeh, Ghahraman Mahmoudi, Dariush Moslemi, Ali Bijani, Mohammad Ali Jahani

**Affiliations:** 1 Babol University of Medical Sciences, Babol, Iran; 2 Hospital Administration Research Center, Sari Branch, Islamic Azad University, Sari, Iran; 3 Department of Radiation Oncology, Babol University of Medical Sciences, Babol, Iran; 4 Social Determinants of Health Research Center, Health Research Institute, Babol University of Medical Sciences, Babol, Iran

**Keywords:** Colorectal cancer, Esophageal cancer, Gastrointestinal cancer, Incidence

## Abstract

**Background:**

Identifying the incidence of cancer helps in planning and prioritizing resources for its screening, prevention, treatment and diagnosis. This study aimed at investigating a 25-year trend in gastrointestinal cancer in northern Iran during 1991-2016.

**Methods:**

This research was a trend analysis. The study population was one thousand five hundred and thirty-five cancer patients referring to Shahid Rajai Hospital in Babolsar, northern Iran, as the only center for radiotherapy in the North of Iran, during 1991-2016. SPSS version 22 was used for entering data and t-test, ᵡ2 and ANOVA were used for analyzing data in the significant level of ≤0.05.

**Results:**

The highest incidence of stomach cancer was 111 (35%) in 2011 and the lowest incidence was 44 (16.3%) in 1996, The highest frequency of esophageal cancer was reported 137(56.1%) cases in 1991 and the lowest frequency was 78 (26.3%) cases in 2016, while the highest incidence of colorectal cancer was 109 (36.7%) cases in 2016 and its lowest frequency was 32 (16.3%) in 1996. There was also a significant difference in the frequency of gastrointestinal cancers in different studied years (p <0.001).

**Conclusion:**

The trends in the incidence of stomach and colorectal cancers in northern Iran were increasing while esophageal cancer was decreasing.

Cancer is a worldwide major problem (1-3) with a considerable economic burden on countries (4, 5). The change in trends of cancer incidence and its rate heavily depends on changes in life-environmental risk factors, the methods of preparing and producing food material, genetic differences and cultural and socio-economic conditions (6-9). The rapid change in these patterns results in the epidemiology of cancers (7, 9). 25 out of every thousand people suffer from cancer in Iran(10), Some studies showed that the incidence of some cancers is increasing and that of others is decreasing worldwide (11). Gastrointestinal cancer is one of most prevalent cancers worldwide (12-14) with the highest mortality rate and its incidence is different in the world, especially Asian regions (15). The most incident gastrointestinal cancers with high mortality rates include Stomach, Esophagus and Colorectal cancers (16), their incident rates are different (17, 18). The trend of incidence of stomach cancer is decreasing worldwide (19-22). In European countries, the incidence of esophageal cancer is increasing and so is colorectal cancer among individuals with <50 years old; however, stomach cancer incidence is in decreasing rate (23-26). In Asian regions, the incident trends of Stomach, Esophageal and Colorectal cancers are in increasing rates with very different epidemiology (27). Siegel et al. found that the incidence of colorectal cancer was in increasing rate in the USA (16) and Joliet et al. reported a similar trend in gastric cancer in Switzerland (23). Based on some studies on cancer incidence among Iranian men and women, the incidence of gastrointestinal tract cancers, as the most prevalent cancers after breast cancer, is increasing (28, 29).

Baniasadi et al. found the increased incidence of colorectal cancer in Southeast Iran during the past decade (30). In Eastern Azerbaijan, the incidence of colorectal cancer is on the rise, but esophageal cancer is declining (31). Cancer is the second most common cause of non-accidental deaths in Iran (32-34). Having appropriate information on trends of cancers is necessary for estimating their economic burden (4). This can help in resource management, resource priorities and cost evaluation for future prevention, treatment and screening those at risk (35, 36). Having this in mind and considering the importance of cancer statistics, trends and its incidence and mortality rates and patterns, this study aimed at investigating the pattern of gastrointestinal cancers in northern Iran during 1991-2016. 

## Methods

This research is a trend analysis. Research population included cancer patients referring to Shahid Rajai Hospital in Babolsar, northern Iran, as the only center for radiotherapy in the north of Iran. A multi-staged random sampling method was used for sampling. At first, studied years were determined by applying a systematic random sampling with the coefficient of five (years 1991, 1996, 2001, 2006, 2011, 2016) for a 25-year period. Then, all 1535 patients referring to the center for the first time for radiotherapy or chemotherapy in the selected years were included in the study. Data collection instrument was a researcher-made checklist validated by consulting 8 professionals. The checklist included information such as patient’s first name and family name, gender, occupation, marital status, the city or state of residence, and either in urban or rural regions. The protocol of study was approved by the Ethics Committees of Babol University of Medical Sciences and Sari Islamic Azad University (IR.IAU.SARI.REC.1396.39). Data were collected from the archived records and the hospital health information management system. Data were analyzed by SPSS Version 22, using the t-test, χ2 and ANOVA in the significant level of ≤0.05. 

## Results

The total number of patients who referred to the center for the first time in the studied years was 1535. Their mean ages of those who firstly develop esophageal cancer, gastric cancer and colorectal cancer were 64.9±13.11, 60.66±12.78 and 55.96±14.80 years old, respectively. There was a significant relationship between the kind of gastrointestinal cancers and age (p<0.001). The most frequent cancer among male and female patients was esophageal cancer with 256 (44.2%) and 372 (39%), respectively (table 1).

**Table 1:** The states of cancer patients based on their demographic and background variables

**Variable **	**Esophageal** **N=628** **N (%)**	**Stomach** **N=464** **N (%)**	**Colorectal** **N=443** **N (%)**	**P value**
Male Female	256(44.2) 372(39)	144(24.8) 320(33.5)	180(31) 263(27.5)	0.002
Urban Rural	829(37.2) 330(33)	248(30.9) 216(29.5)	256(31.9) 187(25.5)	0.003
1991 1996 2001 2006 2011 2016	137(56.1) 117(59.7) 110(53.7) 96(34.8) 90(28.4) 78(26.3)	67(27.5) 47(24.0) 44(21.5) 85(30.8) 111(35.0) 110(37.0)	40(16.4) 32(16.3) 51(24.9) 95(34.4) 109(36.7) 109(36.7)	<0.001

As table 2 shows, the overall mean age of the patients was 61.03±14.01, with the highest and lowest means of 57.84±13.81 in 1991 and of 63.83±14.12 in 2016, respectively. The mean age rates of patients were significantly different in various years with an increasing trend in time (p<0.001). 

**Table 2:** The mean age rates of studied cancer patients during 1991-2016

**Year**	**Case**	**SD** **± ** **Mean**	**95% Confidence Interval for Mean**		**Minimum**	**Maximum**
			**Lower Bound**	**Upper Bound**		
1991	244	13.81 ± 57.84	56.10	59.58	6	94
1996	195	59.85±14.96	57.74	61.97	9	90
2001	204	61.11±13.87	59.19	63.02	26	90
2006	276	61.86±13.75	60.23	63.48	20	89
2011	317	60.84±13.27	59.38	62.31	22	88
2016	297	63.83±14.12	62.22	65.44	11	91
Total	1533	61.03±14.01	60.33	61.74	6	94

P=0.001

**Figure 1:**
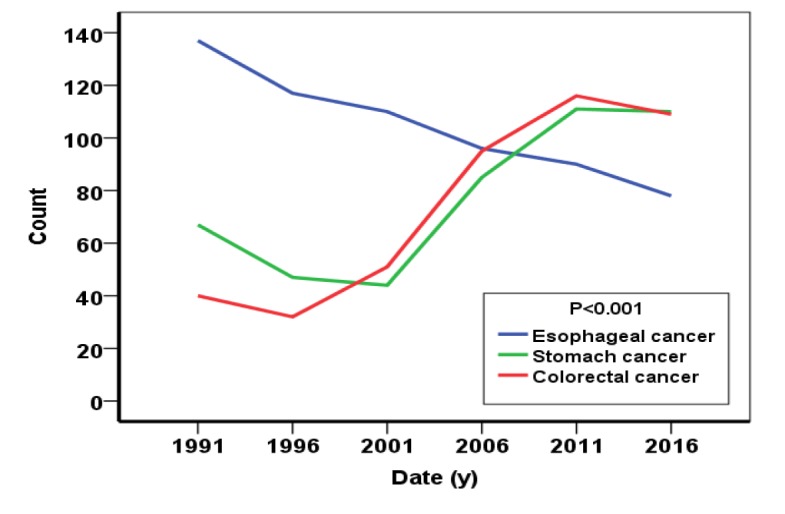
The trends of gastrointestinal cancers between 1991-2016 based on the frequencies of patients referring to the hospital

Figure 1 shows the incidence rates of gastrointestinal cancers among the patients in the selected years. As it can be seen, the patterns of stomach and colorectal cancers showed an increasing trend. The pattern of esophageal cancer, however, showed a decreasing mode. Also, the highest incidence of stomach cancer was reported 111 (35%) in 2011 and the lowest frequency was 44 (16.3%) in 1996, The highest frequency of esophageal cancer was reported to be 137 (56.1%) in 1991 and the lowest frequency was 78 (26.3%) in 2016, while the highest incidence of colorectal cancer was 109 (36.7%) in 2016 and the lowest frequency was 32 (16.3%) in 1996. There was also a significant difference in the frequency of gastrointestinal cancers in different years (p <0.001).

The trends of incidence of esophageal cancer in urban regions and among rural men were decreasing while for the rural women was increasing. However, incidence cancer trends of stomach and colorectal were increasing in rural and urban regions as well as among the rural and urban men and women. 

**Figure 2:**
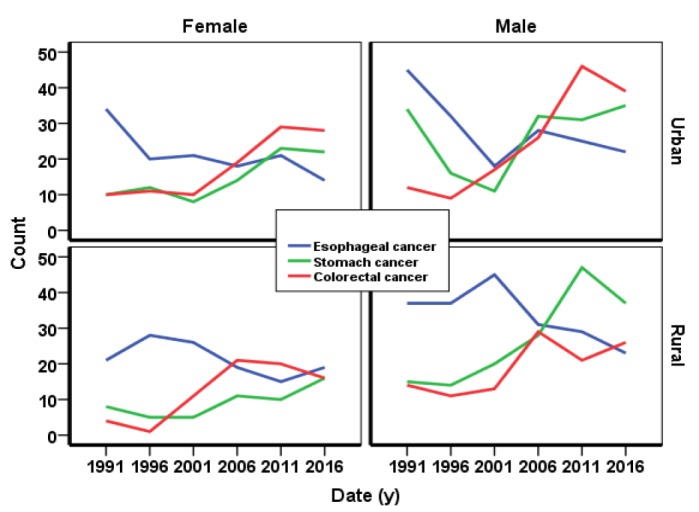
The trend of incidence of gastrointestinal cancers in northern Iran among the rural and urban regions and rural and urban men and women

The speed of incidence of colorectal cancer in urban regions was more than the rural regions (figure 2).

**Figure 3:**
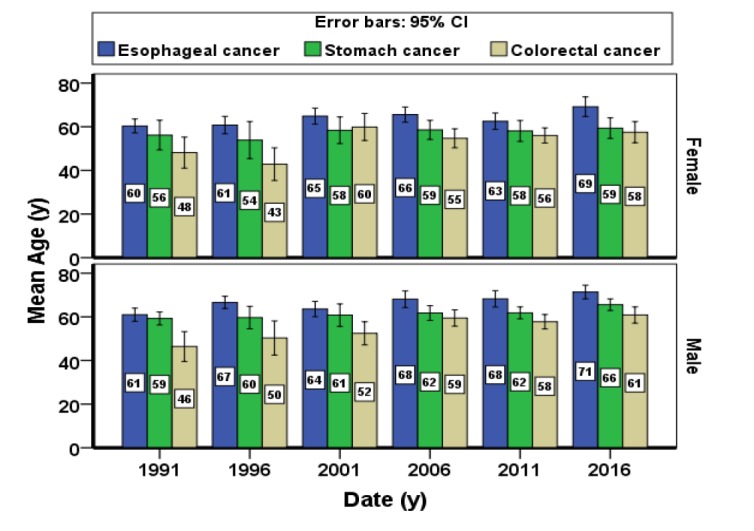
The mean age of patients referring to the hospital center based on the studied year, gender and cancer type during 1991-2016

The mean age of those suffering from esophageal cancer in both male and female patients was higher (65.90±13.11) than that those with stomach cancer (60.66±12.78) and colorectal cancer (55.96±14.80), with an increasing pattern in all studied gastrointestinal cancers (figure 3).

## Discussion

Findings showed that the trends of incidence of stomach and colorectal cancers in northern Iran were increasing and esophageal cancer was decreasing. However, the mean age of patients with gastrointestinal cancer decreased in the studied years. The incidence rate of gastrointestinal cancers in men was significantly higher than that of women. This is in line with the findings of study by Fitzmaurice in which 29 cancer groups in the Eastern Mediterranean had been studied in the years 2005-2015 (35). Arnold et al. in 2015 studied the incidence rate of five common cancers in European countries from 1998 to 2008 and found that the incidence of stomach cancer among men was higher than the women, but with similar trends in the incidence of rectum and colon cancers in men and women (20). 

The incidence of esophageal cancer in this study had a decreasing trend. This cancer is more prevalent in Asia (37), especially in northern Iran (29). In addition, the rate of its incidence among men was higher than the women and the older ones suffered more from it. Joliet et al. found a similar trend in the incidence of gastrointestinal cancers in Switzerland with that of other European countries, where the incidence of esophageal and colorectal cancers was in a considerable increasing trend and stomach cancer was in a decreasing trend (23). 

The incidence of esophageal and gastric cancers in the United States in 1997-2014 has been examined and the findings have shown that the incidence of esophageal cancer in the country has been declining (38). The incidence of esophageal cancer and its relation with human development indicators in 41 countries, including Asian countries showed that the incidence of this cancer is in a decreasing trend, possibly due to changes in lifestyles, population settings and food consumption patterns as well as epidemiological phenomena (39). 

Other studies showed that the incidence of esophageal cancer in different regions has different decreasing-increasing trends (40-42), possibly due to various lifestyles in the world. In the present study, the incidence of esophageal cancer has increased with increasing age, and Thrift's study in the United States and Sweden has also shown an increase in age-related esophageal cancers (43).

Our findings showed that the incidence trend of stomach cancer with a higher rate in Eastern Asia (27) is increasing with a slower slope than that of colorectal cancer in northern Iran. Its incidence was higher in urban regions than the rural areas. Jemal et al. reported a decreased trend in stomach cancer worldwide (7). 

Stating that the pattern of cancer incident is different worldwide and related to human development indicators, Vineis et al. reported a decreasing trend in the incidence of stomach cancer worldwide (1). 

Arnold et al. reported that the incidence of stomach cancers in European countries was in a decreased pattern, mainly due to decreased infection with H.pylori as the main factor of stomach cancer (20). 

The incidence trend of stomach cancer in some Asian countries, such as Japan, China, Colombia, Ukraine and Russia was reported as in a decreasing trend, due to less dependence on salty food and more use of fresh vegetables and fruits (7). This is not the case of our finding, possibly due to administering a comprehensive program for screening and preventing cancers as well as controlling H.pylori in these Asian countries other than Iran. Another possible reason is the difference in dietary intake and the use of more agricultural pesticides in northern Iran.

Our study showed that the incidence rate of colorectal cancer with the highest incidence rate in Eastern Asia and Europe (20) is in a speedy trend in northern Iran in comparison with other gastrointestinal cancers. Ferlay et al reported that this cancer is more common worldwide (9) and its incidence varies widely across regions of the world, reflecting socio-economic development and rising in countries such as Russia, China and Brazil, Canada, the United Kingdom, Denmark and Singapore (44). This cancer is more common in regions with high human development indicators comparing with those with moderate human development indicators, where stomach cancer is more common (11). 

Arnold et al. found colorectal cancer as one of the four common cancers in European countries with an increasing trend in its incidence (20). The increasing trend in the incidence of colorectal cancer can be a sign of dietary pattern and lifestyle changes worldwide (7, 20). Similar studies were conducted in northeastern Iran 2007-2011 (2) and southeast of Iran during 2003-2013 (30), and the results indicate an upward trend in colorectal cancer in these areas.

As another finding, the highest incidence of colorectal cancer belonged to urban regions, conformed the argument by Olrich et al. showing a significant relationship between the incidence of colorectal cancer and dietary patterns (17). It appears that the higher incidence of colorectal cancer in urban population results from changes in their dietary patterns and the use of more synthesized food. 

In conclusion, the incidence of esophageal cancer in northern Iran was in a decreasing trend stomach and colorectal cancers were in increasing trends. The trends can be appropriately managed by making regular screening programs, promotion of community health level, increasing public awareness about cancer and its symptoms, decreasing risky behaviors closely associated with cancer incidence as well as controlling H.pylori, fatness, and changing lifestyle. 
